# The Intricacies of Survivor's Guilt: Exploring Its Phenomenon Across Contexts

**DOI:** 10.7759/cureus.45703

**Published:** 2023-09-21

**Authors:** Karlyle Bistas, Ramneet Grewal

**Affiliations:** 1 Behavioral Health, Wayne State University Detroit Medical Center, Detroit, USA; 2 Medicine, Saba University School of Medicine, The Bottom, NLD

**Keywords:** trauma-focused psychological therapy, cognitive behavioural therapy (cbt), psychological burden, trauma, survivor’s guilt

## Abstract

Survivor’s guilt is the response to an event that some people experience when they survive a traumatic event or situation that others did not. This psychological phenomenon can be associated with post-traumatic stress disorder (PTSD), anxiety disorders, and complicated grief. Survivor’s guilt is described as the manifestation of tension, distress, or solitude that is triggered by a celebration of life, references to the deceased, and expressions of sympathy. Survivor’s guilt is difficult to treat, and the treatment will depend on the severity of the patient’s symptoms and the individual’s specific needs. This symptom is often overlooked, as it gets grouped into the broader recognized disorder of PTSD. It has been studied within various contexts, including instances of multiple causality, bereaved populations, and HIV-positive men.

## Introduction

Survivor's guilt is an intricate psychological phenomenon that has received considerable attention in the domain of mental health. Dr. Stanley Cobb and Dr. Erich Lindemann introduced this term in 1943 [1]. Guilt can be characterized either as dispositional and enduring or as context-dependent and temporary, as observed in guilt associated with traumatic experiences. Studies indicate that guilt linked to trauma is notably widespread among individuals who have experienced traumatic events, and it is associated with heightened psychopathological symptoms and thoughts of suicide [2]. It appears that the first documentation of survivor’s guilt was described in psychoanalytic writings about Holocaust and Hiroshima survivors [3,4].

This psychological phenomenon can emerge in individuals who have encountered or witnessed death and managed to survive the experience [5]. This could result in emotional turmoil and guilt. It has been observed that those who survive may assume culpability for the demise or harm of others, even if they held minimal actual control or impact over the circumstances [5]. Survivor’s guilt was once considered a symptom of post-traumatic stress disorder (PTSD) in the Diagnostic and Statistical Manual of Mental Disorders, Third Edition (DSM-III). This was intended to reflect the research conducted on Vietnam War veterans, who had increased levels of survivor guilt [5]. It was identified as a linked symptom to PTSD in the Diagnostic and Statistical Manual of Mental Disorders, Fourth Edition, Text Revision (DSM-IV-TR), but it was subsequently removed from the Diagnostic and Statistical Manual of Mental Disorders, Fifth Edition, Text Revision (DSM-V-TR). Survivor guilt is now associated with the mood-related symptoms of PTSD. Despite its previous diagnostic significance, there has been limited systematic examination of this phenomenon.

This case report focuses on a patient with survivor’s guilt and the approaches that were taken to improve their psychological well-being. Survivor’s guilt does not have to be in the context of PTSD. Other events that can cause survivor’s guilt include mass shootings, natural disorders, or car accidents. It is important to comprehend this in order to avoid excluding individuals who may not meet the diagnostic criteria for PTSD.

## Case presentation

Subjective

The patient is a 52-year-old Caucasian male with a history of depression, anxiety, and PTSD. He has obstructive sleep apnea (OSA) but does wear his CPAP machine nightly. The patient presented for evaluation on an outpatient basis due to worsening insomnia. Throughout the interview, the patient was pleasant and forthcoming with information. He denies any substance use history. He reports difficulty with both falling and staying asleep. He reports situational anxiety. His anxiety is worse in big crowds and when around airplanes and helicopters. He denies access to firearms. He denies manic symptoms. He reports being depressed since he was a young child. He denies auditory hallucinations. He denies active suicidal ideation, intent, or plan. When asked about visual hallucinations, the patient was tearful and mentioned he often sees his friend who died while overseas in front of him during combat. He reports his goals are for improved mood and sleep. The patient reports frequent, nighttime awakenings associated with nightmares. The patient reports improved sleep while on trazodone. He was in the military and was involved in combat. He reports extreme anxiety surrounding birthdays and celebrations, reporting guilt for being able to experience them. 

Objective

On mental status examination, the patient appeared to be his stated age. He was appropriately groomed and had good hygiene. The patient spoke in low volume with a normal cadence, but his tone was soft. He exhibited spontaneous speech and was fluent in English. The patient’s behavior was calm, cooperative, and pleasant. The patient described his mood as “okay.” The patient displayed dysthymic affect with appropriate range and depth. The patient’s thought process was linear, logical, and coherent. The patient’s thought content was ruminative on guilt and poor sleep. The patient’s judgment and insight were fair, as demonstrated by their understanding of the need for treatment and adherence to psychotropic medications. The patient was awake, alert, and oriented to person, place, and time. The patient’s memory appeared intact for the purposes of the conversation. The patient’s fund of knowledge was adequate. The patient’s vital signs were within normal limits. The patient denied using tobacco, and the Alcohol Use Disorders Identification Test (AUDIT-C) assessment yielded negative results.

The patient’s labs were non-contributory and his urine drug screen (UDS) was negative. The patient denied having any family history of mental illness or suicide. The patient had previously trialed quetiapine (unknown dose or duration) for sleep, doxepin (unknown dose or duration/discontinued due to weight gain) for sleep, venlafaxine (unknown dose or duration) for mood, sertraline (unknown dose or duration) for mood, and bupropion (unknown dose or duration) for mood. The patient was currently on bupropion xl 300 mg in the morning for mood and trazodone 200 mg at bedtime for insomnia. He still has difficulty sleeping on trazodone 200 mg due to ruminative thoughts.

Assessment

The patient was a 52-year-old Caucasian male with a history of depression, anxiety, and PTSD. He had been experiencing depression since he was a young child. The presence of this condition may suggest a biological vulnerability to emotional distress, although the patient did not have a strong family history of mental illness. The patient’s traumatic experiences, such as experiencing combat and witnessing his friend die in front of him, predisposed him to mental disorders. Witnessing the passing of his friend during combat had contributed to worsening anxiety, insomnia, and dysthymic mood. The patient was often at a generalized heightened sense of arousal but did have protective factors and positive coping mechanisms, which indicated a favorable prognosis. The patient was diagnosed with major depressive disorder, recurrent, moderate PTSD, anxiety disorder, unspecified, and insomnia. He was experiencing survivor's guilt as evidenced by the patient having constant thoughts of not doing enough, flashbacks, and guilt when celebrating birthdays.

Plan

The patient was enrolled in therapy and continued his current psychiatric medication regimen. The patient was on bupropion xl 300 mg in the morning for mood and trazodone 200 mg at bedtime for insomnia. He continued to be safe for outpatient management. He was not amenable to trying other medications at this time. He will continue on his current medication regimen and was amenable to starting cognitive behavioral therapy on a weekly basis. His symptoms were improving slowly through CBT as evidenced by improved sleep and mood, and decreased thoughts of guilt. The patient's improvement was based on subjective accounts and symptomatic improvement.

## Discussion

Risk factors and symptoms of survivor’s guilt

Survivor’s guilt is associated with natural disasters, military service, or other traumatic events. There appear to be several risk factors of survivor’s guilt, including closeness to the deceased or witnessing suffering. A core belief in survivor’s guilt is the belief that enough was not done to help or that the person is not enough [6]. These feelings are more likely in people who fear social interaction, confrontation, or rejection [6]. Introversion and low self-esteem may increase the chances of experiencing survivor’s guilt [6]. There are also many symptoms associated with survivor’s guilt such as flashbacks, irritability, social isolation, and nightmares. Other contributing factors are connected to the experienced event and the extent of witness trauma [5]. Survivor guilt may be more probable when there has been a significant number of fatalities since this will intensify the sense of inequity, a core theory in survivor guilt [5].

Other mental disorders associated with survivor’s guilt 

Witnessed trauma is not the only requirement for survivor’s guilt. It can be seen in conditions such as complicated grief, anxiety, and PTSD. It can also manifest in individuals who have not witnessed or experienced trauma [6]. The experience is individualized, can be influenced by various factors, and is often tied to the emotions of responsibility and unfairness [6]. Major depressive disorder may be a consequence of those experiencing survivor’s guilt. Additionally, anxiety disorders may be a predisposing factor or consequence of this psychological phenomenon, as a heightened arousal stage may amplify situations [6]. It can also be observed in obsessive-compulsive disorder (OCD) in the form of intrusive thoughts. Substance use disorders may also be a consequence of the guilt itself. Survivor’s guilt appears to be a symptom of, as well as, a root cause for many mental illnesses. Survivor's guilt is an emotion that isn't always indicative of a mental disorder, yet it seems to be linked with several of them [6].

Review of current literature 

Survivor’s guilt was studied in a United Kingdom stress clinic sample [4]. In this study, people were systematically assessed for survivor guilt over an 18-month period [4]. The results found that 38.5% of participants had survived an event that others did not and that 90% of the survivors experienced the psychological burden of survivor’s guilt [5]. It was determined that surviving a fatal traumatic event was associated with higher levels of suicidality [5]. It should be noted that survivor’s guilt has been studied mostly in observation studies and not empirically tested [5].

There is a strong link between suicidality and survivor guilt [7]. Researchers have found that there is a relationship between survivor's guilt and suicide attempts, as it appears that surviving a fatal traumatic event was associated with suicidality [5,8]. This study also examined intensive combat-related guilt and its association with PTSD. They determined the greater need for clinical attention to the role of guilt in the evaluation and treatment of suicidal veterans with PTSD [7]. Overall, there is a scarcity of research on this topic. Given its apparent association with numerous mental disorders, further research into this phenomenon is warranted. Survivor’s guilt has been associated with more severe PTSD [5,8]. Survivor’s guilt is often seen in veterans who were exposed to traumatic events or those who experienced combat. Survivor’s guilt is an emotion often experienced by those suffering from PTSD [5].

Associations with neuroanatomy 

There has been an association between various brain structures and how these neuronal pathways influence guilt. Functional magnetic resonance imaging (fMRI) has been used in healthy individuals to identify specific brain regions associated with guilt [8]. Compared with the control groups, it was found that guilt episodes recruited a region of the right orbitofrontal cortex, which has been highly correlated with individuals’ propensity to experience guilt [6]. In addition, "guilt-specific" neuronal activity was observed in the paracingulate dorsomedial prefrontal cortex [8]. This region of the brain is the same region of the brain known as the “Theory of Mind” region, which provides a new awareness of the nature of guilt as a conscious moral emotion [8]. Interestingly, this new insight can link a neural base to antisocial disorders [9]. Survivor’s guilt could involve heightened activity or differences in these brain structures. There are many other brain structures known to play a role in the guilt and emotional processing of trauma. fMRI has shown a heightened level of activity in the amygdala in patients with PTSD [7].

Diagnosis and treatment of survivor’s guilt 

Defined in the DSM-IV as ‘guilt about surviving when so many others did not or about things one had to do to survive’, survivor guilt was listed as a symptom of PTSD in the DSM-III as an associated feature of the disorder in DSM-IV [5,8]. Irrespective of the incident that gives rise to this phenomenon, it can be treated similarly. Despite a high prevalence among traumatized groups, few models have been developed to guide treatment [5]. Further exploration of treatments for individuals who haven't directly witnessed the trauma is warranted. Cognitive behavioral therapy may be used, as it targets the equity theory of survivor’s guilt, which suggests people prefer outcomes that are fair and deserving [5]. Alongside medication management for symptoms, cognitive behavioral therapy (CBT) seems to be beneficial for addressing distorted thinking patterns associated with guilt [10]. Trauma-focused CBT and eye movement desensitization have also shown promising results for those suffering from this [10]. Survivor guilt is a problematic symptom to treat and warrants more research. CBT helps survivor's guilt by focusing on the patient's thoughts and focuses on changing their patterns of shame or self-blame. Practicing gratitude can also help with cognitive distortions [10].

## Conclusions

This case study examines an individual suffering from survivor’s guilt, a psychological phenomenon that is not currently considered a diagnosis in DSM-5-TR. The authors aim to clarify the relationship between survivor's guilt across different contexts and other mental disorders while also outlining approaches to treatment. This emphasized the need for clinicians to differentiate if survivor guilt is associated with or is a symptom of a mental disorder. This is important when providers are making treatment and diagnostic choices. Additionally, the case study brings attention to the shortcomings of the DSM-5-TR and illustrates the significance of maintaining an inclusive perspective when addressing mental health conditions. The study's limitations are that there needs to be more research on how survivor's guilt and PTSD are interrelated on a neuroanatomical level and if treatments for survivor's guilt differ depending on their etiology.
